# mPGES-1/PGE2 promotes the growth of T-ALL cells in vitro and in vivo by regulating the expression of MTDH via the EP3/cAMP/PKA/CREB pathway

**DOI:** 10.1038/s41419-020-2380-9

**Published:** 2020-04-06

**Authors:** Yiqing Li, Jiaoting Chen, Wenjuan Yang, Hongyun Liu, Jieyu Wang, Jie Xiao, Shuangfeng Xie, Liping Ma, Danian Nie

**Affiliations:** 1 Guangdong Provincial Key Laboratory of Malignant Tumor Epigenetic and Gene Regulation, Sun Yat-Sen Memorial Hospital, Sun Yat-Sen University, Guangzhou, China; 2 Department of Hematology, Sun Yat-Sen Memorial Hospital, Sun Yat-Sen University, Guangzhou, China; 3 Department of Hematology, The Sixth Affiliated Hospital, Sun Yat-Sen University, Guangzhou, China

**Keywords:** Acute lymphocytic leukaemia, Acute lymphocytic leukaemia

## Abstract

T-cell acute lymphoblastic leukaemia (T-ALL) is an aggressive haematological malignancy that is characterized by a high frequency of induction failure and by early relapse. Many studies have revealed that metadherin (MTDH) is highly expressed in a variety of malignant solid tumours and plays an important role in the occurrence and development of tumours. However, the relationship between the expression of MTDH and T-ALL has not yet been reported, and the regulatory factors of MTDH are still unknown. Our previous studies found that mPGES-1/PGE2 was important for promoting the growth of leukaemia cells. In the present study, we found that MTDH was highly expressed in primary T-ALL cells and in the Jurkat cell line. Our results showed that mPGES-1/PGE2 regulates the expression of MTDH through the EP3/cAMP/PKA-CREB pathway in T-ALL cells. Downregulation of MTDH inhibits the growth of Jurkat cells in vitro and in vivo. Our results suggest that MTDH could be a potential target for the treatment of T-ALL.

## Introduction

Acute lymphoblastic leukaemia is a highly heterogeneous haematological malignancy with an incidence of approximately one in 100,000 adults^1^. Although very similar regimens are used, the prognosis in patients with T-cell acute lymphoblastic leukaemia (T-ALL) is worse than that in patients with B-cell acute lymphoblastic leukaemia (B-ALL) due to treatment failure and early relapse^2,3^. The survival rates of patients with T-ALL have markedly improved because of the aggressive treatments, including transplantation and new salvage regimens, used in recent decades, but most relapsed patients still experience poor clinical outcomes^4^.

Recent studies have implicated the role of microsomal prostaglandin E synthase-1 (mPGES-1) in several solid tumours, such as breast cancer, prostate cancer and colon cancer^5–7^. mPGES-1 is the terminal synthase responsible for converting cyclooxygenase (COX)-derived prostaglandin H2 (PGH2) into prostaglandin E2 (PGE2). Our previous studies, for the first time, confirmed that mPGES-1 was highly expressed in human leukaemia primary cells and acute myeloid leukaemia cell lines HL-60 and K562. Inhibiting mPGES-1/PGE2 could induce the apoptosis, inhibit the proliferation, arrest the cell cycle and improve the chemosensitivity of leukaemia cells in vivo and in vitro^8,9^. However, the roles of mPGES-1/PGE2 in T-ALL cells are still largely unknown.

MTDH, also named AEG-1 or LYRIC, is an oncogene that was first identified in astrocytes infected with HIV-1 or treated with tumour necrosis factor α^10^. AEG1 mRNA encodes a single-pass transmembrane protein with a predicted molecular mass of approximately 64 kDa, which also colocalizes with ZO1 to form a tight junction complex^11^. MTDH is highly expressed in a variety of malignant tumours and is associated with tumour progression through processes including initiation, proliferation, invasion, metastasis and chemoresistance^12,13^. Studies have also shown that high expression of MTDH is significantly associated with poor prognosis^14^. As a multifunctional mediator of carcinogenesis, MTDH was found to be involved in multiple signalling pathways, such as PI3K/Akt, NF-κB, Wnt/β-catenin and MAPK^15–17^. Whether MTDH has a similar role in T-ALL cells and what molecular regulatory mechanism is involved remain poorly understood.

In the present study, we found that mPGES-1 and MTDH are highly expressed in T-ALL primary cells and Jurkat cells and that downregulation of mPGES-1 inhibits MTDH expression via the EP3/cAMP/PKA-CREB pathway. MTDH knockdown inhibits the proliferation of T-ALL cells in vitro and in vivo.

## Materials and methods

### Materials

The human T-ALL cell line Jurkat and human *embryo kidney cell line 293T* (*HEK293T*) *were* obtained from the Hematology Research Institute (Tianjin, China). Normal mononuclear cells were separated from the peripheral blood of healthy volunteers, and primary T-ALL cells were separated from the peripheral blood of three T-ALL patients with their consent (all experiments involving volunteers and patients were approved by the ethics committee of Sun Yat-Sen Memorial Hospital). Athymic nu/nu mice (4 weeks old) were obtained from the laboratory animal centre of the east campus of Sun Yat-Sen University (all animal experiments were conducted in strict compliance with institutional guidelines). The anti-mPGES-1 antibody (10004350), mPGES-1 inhibitor CAY10526 (10010088), exogenous PGE2 (14010), EP1 receptor inhibitor SC-19220 (14060), EP2 receptor inhibitor AH-6809 (14050) and EP4 receptor inhibitor L-161982 (10011565) were purchased from Cayman Chemical Company (Ann Arbor, MI, USA). The EP3 receptor inhibitor L-798106 (L4545) was purchased from Sigma-Aldrich Corp. (St. Louis, MO, USA). The adenylate cyclase (AC) agonist forskolin (S2449) and the protein kinase A (PKA) inhibitor H89 (S1582) were purchased from Selleck Chemical Company (Shanghai, China). The adenosine 3′5′-cyclic monophosphate (cAMP) parameter assay kit (KGE002B) was purchased from R&D Systems, Inc. (Minneapolis, MN, USA). The anti-EP1 receptor (ab217925), anti-EP2 receptor (ab167171), anti-EP3 receptor (ab21227), anti-EP4 receptor (ab45295), anti-MTDH (ab124789), and anti-Ki67 (ab16667) antibodies were purchased from Abcam Trading Ltd. (Shanghai, China). Anti-CREB (9197), anti-p-CREB (9198), and anti-GAPDH (5174) antibodies were purchased from Cell Signaling Technology, Inc. (Danvers, MA, USA).

### Cell culture

Cells were cultured in RPMI 1640 medium containing 10% foetal bovine serum (both Gibco; Thermo Fisher Scientific, Inc., Waltham, MA, USA; 31870082, 10100147) at 37 °C in 5% CO_2_. Peripheral blood mononuclear cells (PBMCs) were separated by Ficoll-Hypaque gradient.

### Immunofluorescence analysis

For the detection of EP receptors, Jurkat cells were plated on glass coverslips. After fixing with 4% paraformaldehyde for 30 min at room temperature and blocking with 5% bovine serum albumin (BSA) in phosphate buffer saline (PBS) containing 0.1% Triton X-100, the cells were incubated with the EP1/EP2/EP3/EP4 receptor antibody (diluted according to the instructions), followed by goat anti-rabbit IgG Cy3 (Abcam; ab6939) for 1 h. Then, the cells were washed with PBS three times and stained with 4',6-diamidino-2-phenylindole (DAPI) (Abcam; ab104139) for 30 min. Fluorescence images were acquired using a Zeiss LSM 800 Confocal Imaging System.

### Western blot analysis

Cells were lysed with an appropriate volume of radioimmunoprecipitation buffer supplemented with protease and phosphatase inhibitor cocktails, and the protein concentrations were determined by bicinchoninic acid assays with BSA (all CWBIO; CW2200S, CW2383, CW0017) as the standard. A total of 30 ng/20 µl protein was separated by 10% SDS-PAGE and transferred to polyvinylidene difluoride membranes (EMD Millipore, Billerica, MA, USA; C3117). Following blocking with Tris-buffered saline containing 5% BSA diluted in TBS with Tween-20 for 1 h, the membranes were incubated overnight at 4 °C with primary antibodies diluted according to the instructions, followed by incubation with horseradish peroxidase-conjugated secondary antibodies for 1 h at room temperature. The immunoreactive bands were detected using a chemiluminescence system (Thermo Fisher Scientific, Inc., Waltham, MA, USA) and quantified using ImageJ 1.43 (National Instituted of Health, Bethesda, MD, USA).

### Dual-luciferase reporter assay

The transcript of human gene MTDH (NM_178812) was downloaded from National Center for Biotechnology Information (NCBI). The fragment containing MTDH promotor (−1400 bp to +100 bp) was selected for designing suitable primers and subsequently cloned into pGL3-basic vector, named as pGL3-MTDH. HEK293T cells were cultured in six-well plates and cotransfected with plasmid pCMV-GFP-Puro-01-CREB1 (750 ng/well, pCMV-GFP-Puro-NC as negative control) and reporter plasmid (pGL3-MTDH (750 ng/well)) with the pRL-TK (all plasmids were purchased from lqbiotech Co., Ltd., Shanghai, China) to establish transfection efficiency. Forty-eight hours after transfection, luciferase activity was measured using a Dual-Glo Luciferase Assay System (Promega Corp., Madison, WI, USA). Relative luciferase activity was calculated by normalizing to the renilla luciferase activity.

### cAMP ELISA

The supernatant of the cell culture was collected, and the concentrations of cAMP in the supernatant were tested by a human cAMP-specific ELISA according to the manufacturer’s instructions.

### Lentivirus infection

Gene knockdown was performed using lentiviral short hairpin RNA (shRNA). The plasmid synthesis and lentivirus packaging were conducted by GenePharma Co., Ltd. (Shanghai, China). The lv-sh-MTDH targeting sequence was 5′-GATTCTGACAAGAGCTCTTCC-3′, and the lv-sh-negative control (NC) targeting sequence was 5′-TTCTCCGAACGTGTCACGT-3′. The lentivirus was added to Jurkat cells in the presence of 5 µg/mL polybrene. Positively transfected cells were selected with 1 µg/mL puromycin after 24 h incubation at 37 °C in 5% CO_2_. Stable cell lines were verified by western blot and RT-PCR analysis.

### Cell proliferation assay

Cell viability was determined using the Cell Counting Kit-8 assay (CCK-8, Dojindo, Kumamoto, Japan). The cells were seeded at 1 × 10^4^ cells/well in 96-well plates and incubated at 37 °C with 5% CO_2_ for 24, 48, and 72 h. The cells were further divided into four groups: (i) KD group, Jurkat cells transfected with lv-sh-MTDH; (ii) NC group, Jurkat cells transfected with lv-sh-NC; (iii) control (CON) group, Jurkat cells without any treatment; (iv) KD + PGE2 group, KD cells cocultured with exogenous PGE2. A total of 10 µl CCK-8 was added to each well and then incubated for an additional 4 h. The optical density (OD) values were measured by using a microplate reader (Bio-Rad Laboratories, Inc., Hercules, CA, USA) at 450 nm. Cell viability was calculated with the formula: cell viability = (dosing OD − blank OD/control cells OD − blank OD) × 100%.

### Flow cytometry

For cell cycle analysis, a total of 5 × 10^5^ cells were fixed with 70% prechilled ethanol overnight at 4 °C and stained with propidium iodide for 10 min at room temperature. The DNA content was analysed by a BD FACStar flow cytometer. For cell apoptosis analysis, a total of 1 × 10^6^ cells were collected and incubated with 5 µl annexin V-fluorescein isothiocyanate (BD Biosciences, Franklin Lakes, NJ, USA; 556454) for 15 min at room temperature in the dark and subsequently with 2.5 µl propidium iodide staining solution (BD Biosciences; 556463). Cells were analysed with a FACScan flow cytometer. Fluorophores were excited at 640 nm. Data acquisition and analysis were performed by using CellQuest software (v6.1×).

### Tumorigenicity of Jurkat cells in nude mice

All athymic nu/nu mice were kept under specific pathogen-free conditions given an autoclaved standard diet and water ad libitum. Eighteen mice were equally divided into three groups. KD group mice were injected with Jurkat cells transfected with lv-sh-MTDH, NC group mice were injected with Jurkat cells transfected with lv-sh-NC, and CON group mice were injected with Jurkat cells without transfection. Cells suspended in PBS were injected subcutaneously into the right flank of the mice. The size of the tumours was measured every 3 days. On the 15th day after injection, mice were euthanized, and tumours were weighed after necropsy. Tumour volume (V) was monitored by measuring the length (L) and width (W) with callipers and calculated with the formula (L × W^2^)/2.

### Histology and immunohistochemistry

Serial 4-μm-thick tissue sections of the tumours were cut, deparaffinized, rehydrated and then heated for 30 min in citrate buffer (pH 6.0) for antigen retrieval. Endogenous peroxidase was inactivated by 3% hydrogen peroxide for 10 min. The sections were then blocked with 5% normal blocking serum before being incubated with anti-MTDH and anti-Ki67 antibodies for 2 h at 37 °C. The sections were then incubated with anti-IgG serum for 20 min at 37 °C. The primary antibodies were visualized over a 10-min period by using a diaminobenzidine tetrachloride kit (ZSGB-BIO, Beijing, China; ZLI-9017). The sections were subsequently observed under a light microscope. All sections were independently assessed and scored by two pathologists.

### Statistical analysis

Data are presented as the mean ± standard deviation (s.d.) of at least three independent experiments. Statistical significance was determined by using one-way analysis of variance followed by Student−Newman−Keuls post hoc tests. *p* < 0.05 was considered to be a statistically significant difference. SPSS 20.0 software (IBM Corp., Armonk, NY, USA) was used for the analyses.

## Results

### mPGES-1 was highly expressed in T-ALL primary cells, and inhibiting mPGES-1 induced apoptosis of T-ALL

**Fig. 1:**
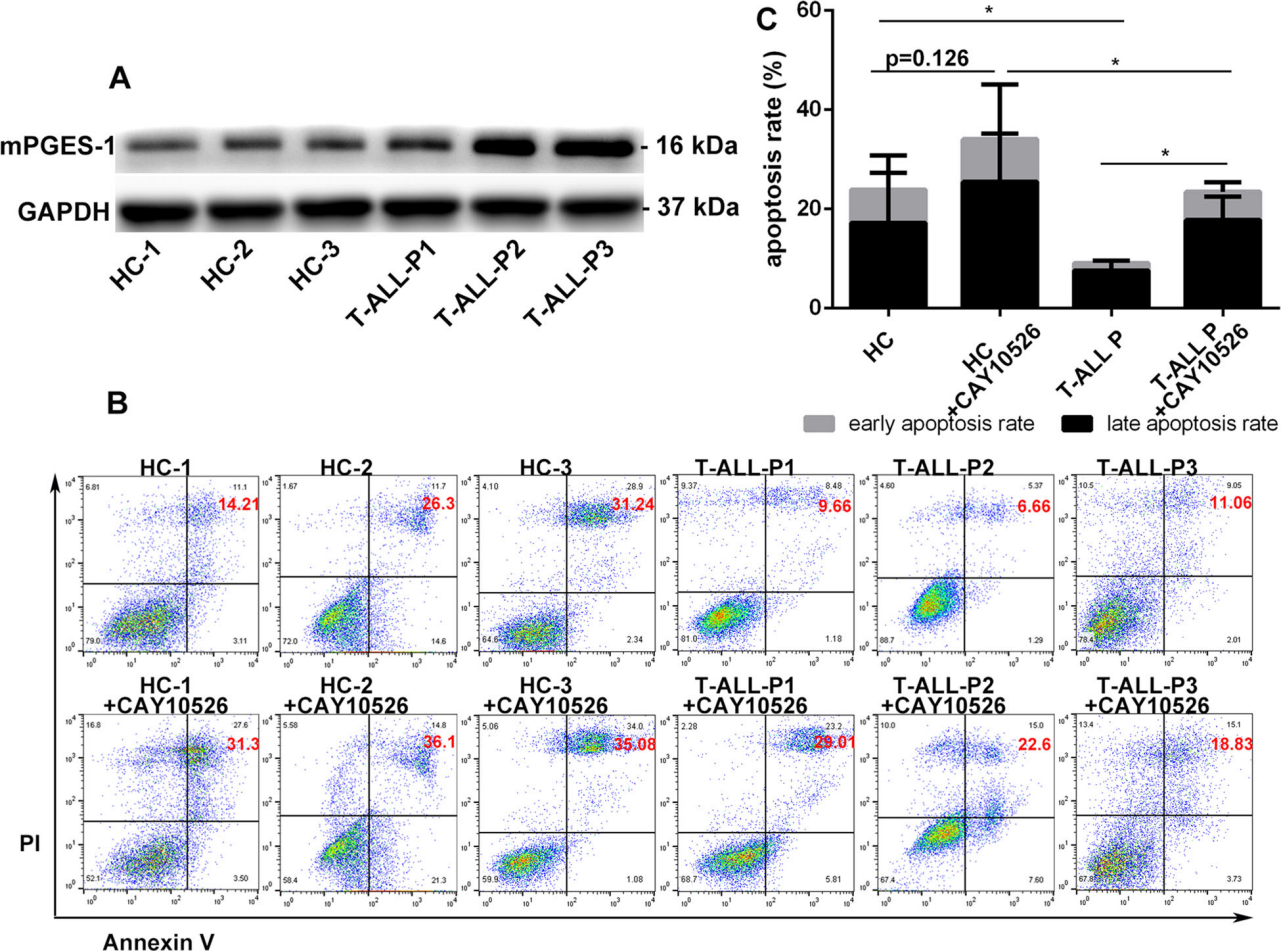
**mPGES-1 was highly expressed in T-ALL primary cells and inhibiting mPGES-1 induced apoptosis of T-ALL.** mPGES-1 expression was higher in T-ALL patient PBMCs than in HC-PBMCs (**a**). CAY10526 (a selective inhibitor of mPGES-1) induced apoptosis in PBMCs from T-ALL patients (**b**, **c**). *p < 0.05.

To investigate the potential role of mPGES-1 in T-ALL, we initially detected the expression of mPGES-1 in PBMCs from healthy control volunteers (HCs) and patients who were newly diagnosed with T-ALL. mPGES-1 expression was significantly higher in PBMCs of T-ALL patients compared with HCs (*p* < 0.05) (Fig. 1a). Next, we checked the effect of CAY10526, a selective inhibitor of mPGES-1, on the apoptosis of these primary cells. After treatment with CAY10526 (16.7 µmol/L) for 24 h, the total apoptosis rate in PBMCs of T-ALL patients significantly increased (9.1 ± 2.2 vs. 23.5 ± 5.1) (*p* < 0.05), while no significant change in apoptosis was found in HC-PBMCs (23.9 ± 8.8 vs. 34.1 ± 2.6) (*p* > 0.05) (Fig. 1b, c). These results indicated that mPGES-1 was highly expressed in T-ALL primary cells and that inhibiting mPGES-1 could induce cell apoptosis.

### MTDH was overexpressed in T-ALL primary cells and could be regulated by mPGES-1/PGE2

**Fig. 2:**
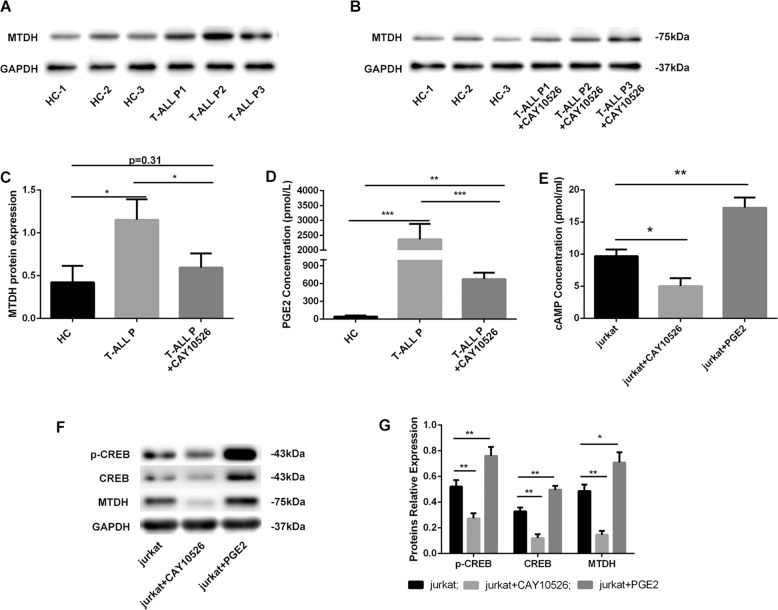
**MTDH was overexpressed in T-ALL primary cells and could be regulated by mPGES-1/PGE2.** MTDH expression was higher in T-ALL patient PBMCs compared with HC-PBMCs (**a**). CAY10526 (16.7 µmol/L, 24 h) reduced PGE2 synthesis and downregulated MTDH expression in primary T-ALL cells (**b**–**d**). The expression of cAMP, p-CREB, CREB and MTDH was downregulated by CAY10526 (16.7 µmol/L, 24 h), and the expression of the above proteins was increased by exogenous PGE2 (50 µmol/L, 24 h) in Jurkat cells (**e**–**g**). ***p < 0.001, **p < 0.01, *p < 0.05.

Studies have shown that MTDH plays an important role in tumour progression by mediating unlimited cell growth, apoptosis avoidance, cell migration and invasion, angiogenesis and drug resistance^18^. Our data showed that the expression of MTDH was higher in the PBMCs of T-ALL patients than in the PBMCs of HCs (*p* < 0.05) (Fig. 2a, c), while CAY10526 (an inhibitor of mPGES-1) significantly inhibited MTDH expression (*p* < 0.05) (Fig. 2b, c) and PGE2 synthesis (*p* < 0.001) (Fig. 2d) in the PBMCs of T-ALL patients. These results suggested that mPGES-1/PGE2 may partially regulate the expression of MTDH.

Our previous study revealed that mPGES-1 is highly expressed in Jurkat cells, the T-ALL cell line^19^. Due to the short survival time of T-ALL primary cells when cultured in vitro, the Jurkat cell line was used as the representative of T-ALL to conduct the following experiments to explore the possible regulatory mechanism of mPGES-1 on MTDH in T-ALL cells. Jurkat cells were treated with mPGES-1 inhibitor (CAY10526) and exogenous PGE2, and the expression of cAMP, p-CREB, CREB and MTDH was measured. As expected, CAY10526 decreased the expression of cAMP, p-CREB, CREB and MTDH, whereas exogenous PGE2 increased the expression of these proteins in Jurkat cells (Fig. 2e–g). These results suggested that mPGES-1 promoted MTDH expression by regulating PGE2 synthesis, and this process might be associated with activation of the cAMP/CREB pathway.

### mPGES-1/PGE2 regulated MTDH expression via the cAMP/PKA-CREB pathway

**Fig. 3:**
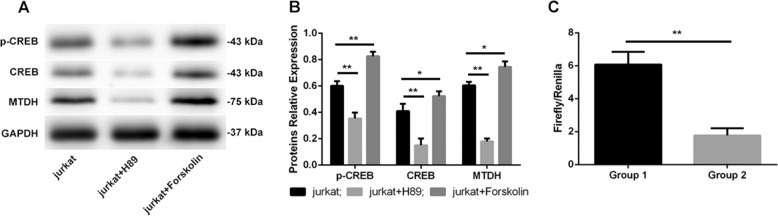
**mPGES-1/PGE2 regulated MTDH expression via the cAMP/PKA-CREB pathway.** The adenylate cyclase (AC) agonist forskolin increased the expression of p-CREB, CREB and MTDH, while the protein kinase A (PKA) inhibitor H89 induced the opposite effects on these proteins (**a**, **b**). The relative luciferase activity (firefly/Renilla) in group 1 (cells cotransfected with pCMV-GFP-Puro-01-CREB1, pGL3-MTDH and pRL-TK) was almost 3.43 times that in group 2 (cells cotransfected with pCMV-GFP-Puro-NC, pGL3-MTDH and pRL-TK) (**c**). **p < 0.01, *p < 0.05.

We further investigated whether the regulatory effect of mPGES-1/PGE2 on MTDH was achieved through the activation of the cAMP/PKA-CREB pathway. Jurkat cells were pretreated with forskolin (25 µmol/L), an adenylate cyclase agonist, and H89 (50 µmol/L), a PKA inhibitor, for 2 h. It was expectedly found that forskolin increased the expression of CREB and MTDH and enhanced the phosphorylation of CREB. However, H89 had the opposite effect on these proteins (Fig. 3a, b). Taken together, these results implied that the changes in MTDH expression caused by PGE2 may be regulated via the cAMP/PKA pathway.

Furthermore, to detect the relationship between CREB and MTDH, a dual-luciferase reporter assay was performed on 293T cells. The results showed that overexpression of CREB could increase the transcriptional activity of MTDH (*p* < 0.01) (Fig. 3c), which suggested that CREB activates MTDH transcription.

### Specific inhibition of the EP3 receptor decreases the expression of cAMP/p-CREB/CREB/MTDH

**Fig. 4:**
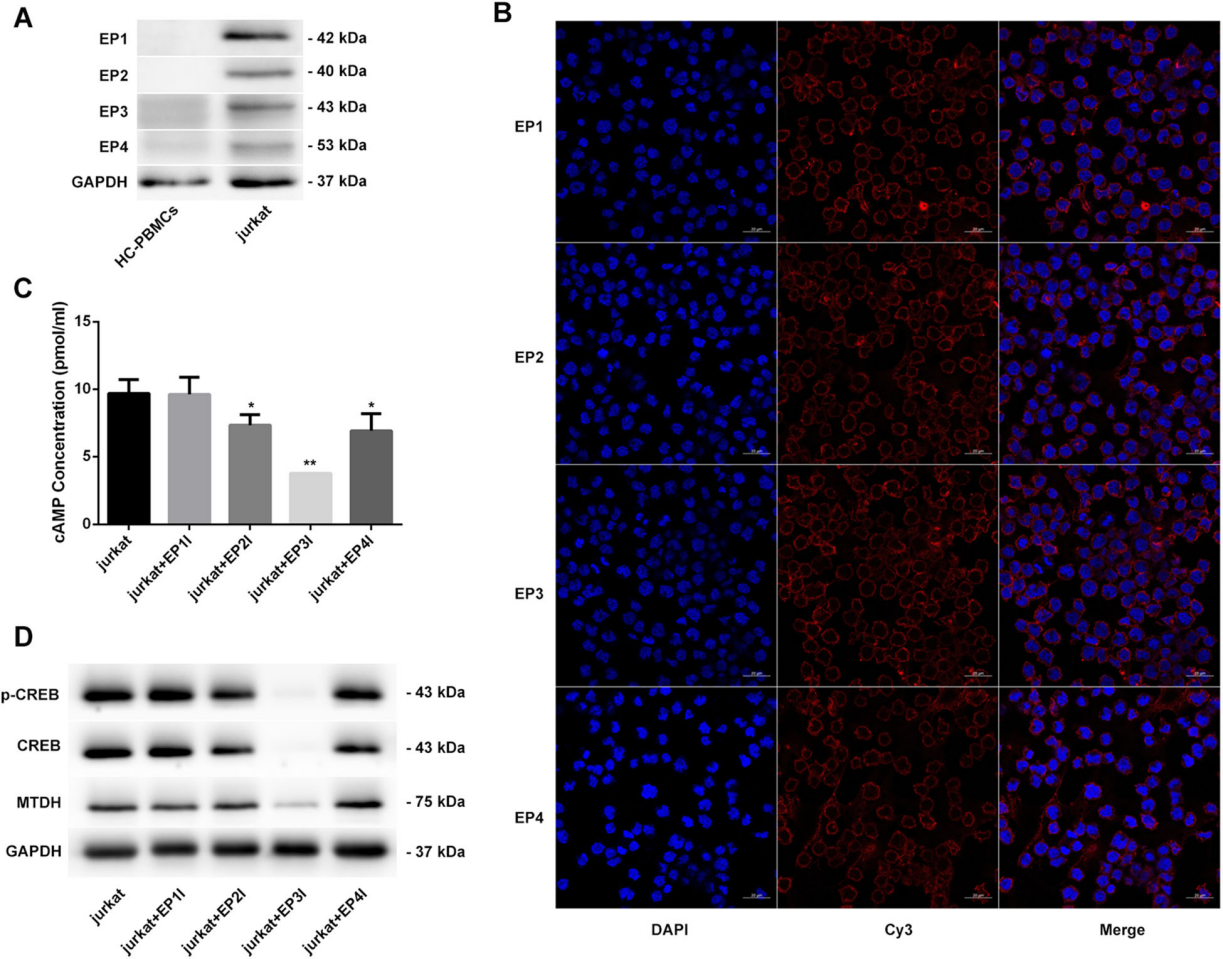
**Specific inhibition of the EP3 receptor decreases the expression of cAMP/p-CREB/CREB/MTDH.** All four EP receptors were highly expressed in Jurkat cells and mainly located at the cell membrane (**a**, **b**). Jurkat cells were cocultured with EP1I (100 µmol/L), EP2I (200 µmol/L), EP3I (100 µmol/L) and EP4I (33.3 µmol/L) for 24 h; EP1I had no effect on the expression of cAMP, p-CREB and CREB, while EP2I, EP3I and EP4I reduced the expression of cAMP, p-CREB and CREB, and EP3I had the most significant effect. Only EP3I induced a significant decrease in the expression of MTDH (**c**, **d**). **p < 0.01, *p < 0.05, compared with Jurkat cells.

A number of studies have shown that PGE2 exerts its function mainly via four subtypes of G-protein-coupled receptors, EP1, EP2, EP3 and EP4. In this study, we measured the expression of the four receptors in Jurkat cells by western blotting and immunofluorescence. We found that all four subtypes of EP receptors were highly expressed in Jurkat cells and located at the cell membrane but rarely expressed in HC-PBMCs (Fig. 4a, b). Therefore, the biological function of PGE2 in Jurkat cells may be closely related to all four types of EP receptors.

In the next step, EP receptor inhibitors EP1I SC-19220 (100 µmol/L), EP2I AH-6809 (200 µmol/L), EP3I L-798106 (100 µmol/L) and EP4I L-161982 (33.3 µmol/L) were cocultured with Jurkat cells for 24 h to determine which types of receptors participated in the effect of PGE2 on cAMP, p-CREB, CREB and MTDH. The results showed that EP2I, EP3I and EP4I significantly reduced the concentration of cAMP, especially EP3I, which reduced the concentration of cAMP by almost 61% (*p* < 0.01) (Fig. 4c). Correspondingly, EP2, EP3 and EP4 receptor inhibitors reduced the expression of p-CREB and CREB, whereas EP3 receptor inhibitors significantly reduced the expression of p-CREB and CREB (*p* < 0.01). Furthermore, only EP3 receptor inhibitors, but not EP1, EP2 or EP4 receptor inhibitors, reduced MTDH expression (*p* < 0.01) (Fig. 4d). These data suggested that the effect of PGE2 on the expression of cAMP, p-CREB, CREB and MTDH in Jurkat cells was mediated mainly by the EP3 receptor.

### Silencing MTDH inhibited proliferation, induced apoptosis and arrested the cell cycle of Jurkat cells in vitro

RNA interference (RNAi) was used to examine the tumorigenic effect of MTDH. Jurkat cells were stably transfected with lv-sh-NC (negative control group, NC) and lv-sh-MTDH (knockdown group, KD), and the transfection efficiency was detected by western blotting.

**Fig. 5:**
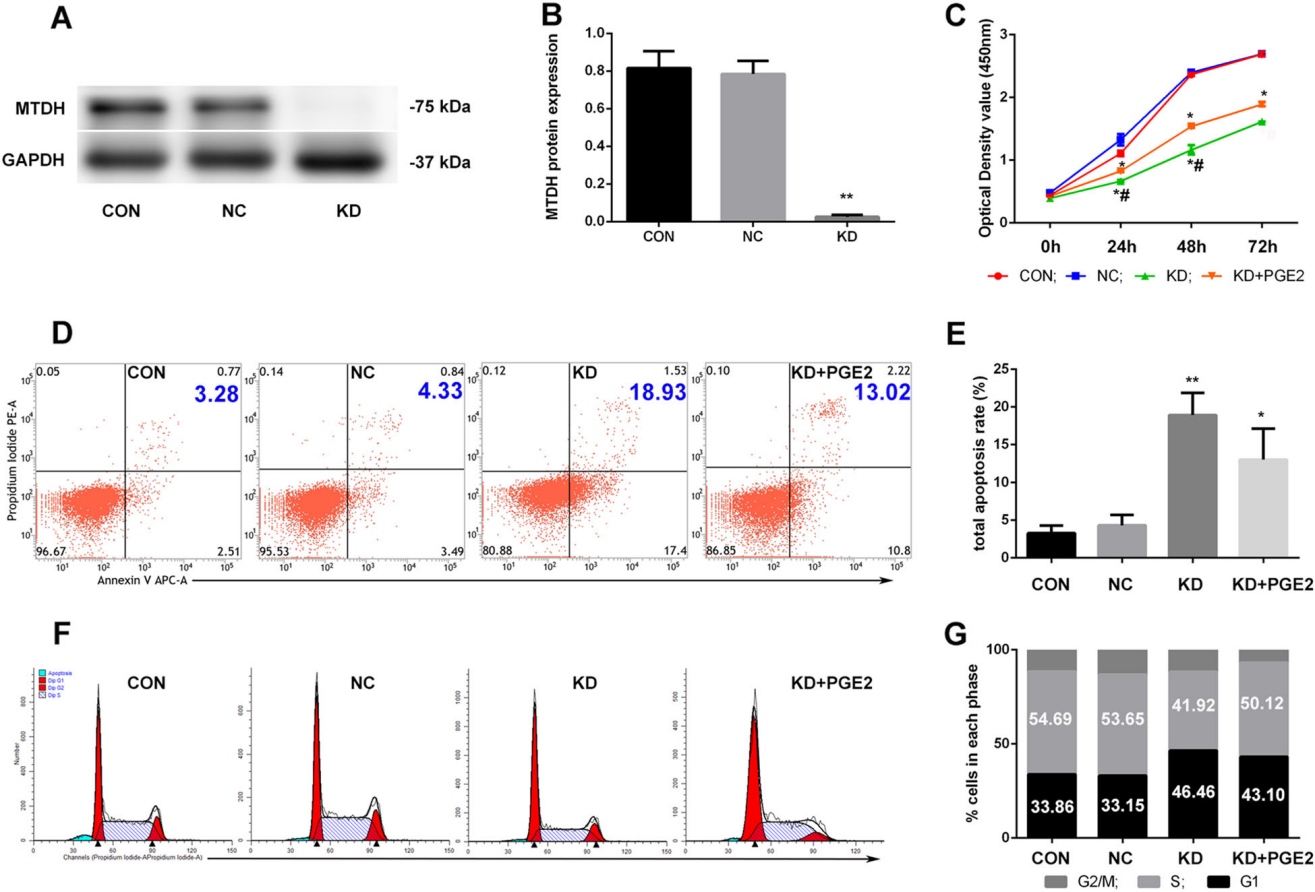
**Silencing MTDH inhibited proliferation, induced apoptosis and arrested the cell cycle of Jurkat cells in vitro.** The expression of MTDH was knocked down nearly 91.7% by shRNA interference (**a, b**), and silencing MTDH inhibited proliferation (**c**), induced apoptosis (**d**, **e**) and arrested more Jurkat cells at G1 phase (**f**, **g**), but these effects were partially attenuated by coculturing cells with exogenous PGE2. **p < 0.01, *p < 0.05, compared with the CON group. ^#^*p* < 0.05, compared with the KD + PGE2 group.

The results showed that the expression of MTDH in the KD group decreased significantly by nearly 91.7% compared with that in the CON group (*p* < 0.01), and no significant difference was found between the CON group and NC group (*p* > 0.05) (Fig. 5a, b), which suggests that gene silencing was effective. Cell proliferation was measured by using the Cell Counting Kit-8 assay. Cell viability in the KD group was much lower than that in the CON group (*p* < 0.05), and coculture with exogenous PGE2 in the KD group partially attenuated the antiproliferative effect of RNA interference with MTDH (*p* < 0.05, compared with the KD group), while no difference was found between the NC group and the CON group (*p* > 0.05) at 24, 48 and 72 h (Fig. 5c). The apoptosis rate and cell cycle were tested by flow cytometry. In the KD group, the total apoptosis rate was much higher than that in the CON group and NC group (*p* < 0.01). In the KD + PGE2 group, the total apoptosis rate was slightly reduced, although not significantly, compared with that in the KD group (Fig. 5d, e). Compared with the CON group and NC group, the KD group had fewer cells in S phase (%) and more cells in G1 phase (%) (*p* < 0.05), while coculture with PGE2 slightly attenuated the changes in the KD group (Fig. 5f, g). Taken together, these results indicate that the effect of PGE2 on Jurkat cells was partially implemented by MTDH and that silencing MTDH could inhibit the proliferation, induce the apoptosis and arrest the cell cycle of Jurkat cells in vitro.

### Silencing MTDH inhibited the growth of Jurkat cells in vivo

**Fig. 6:**
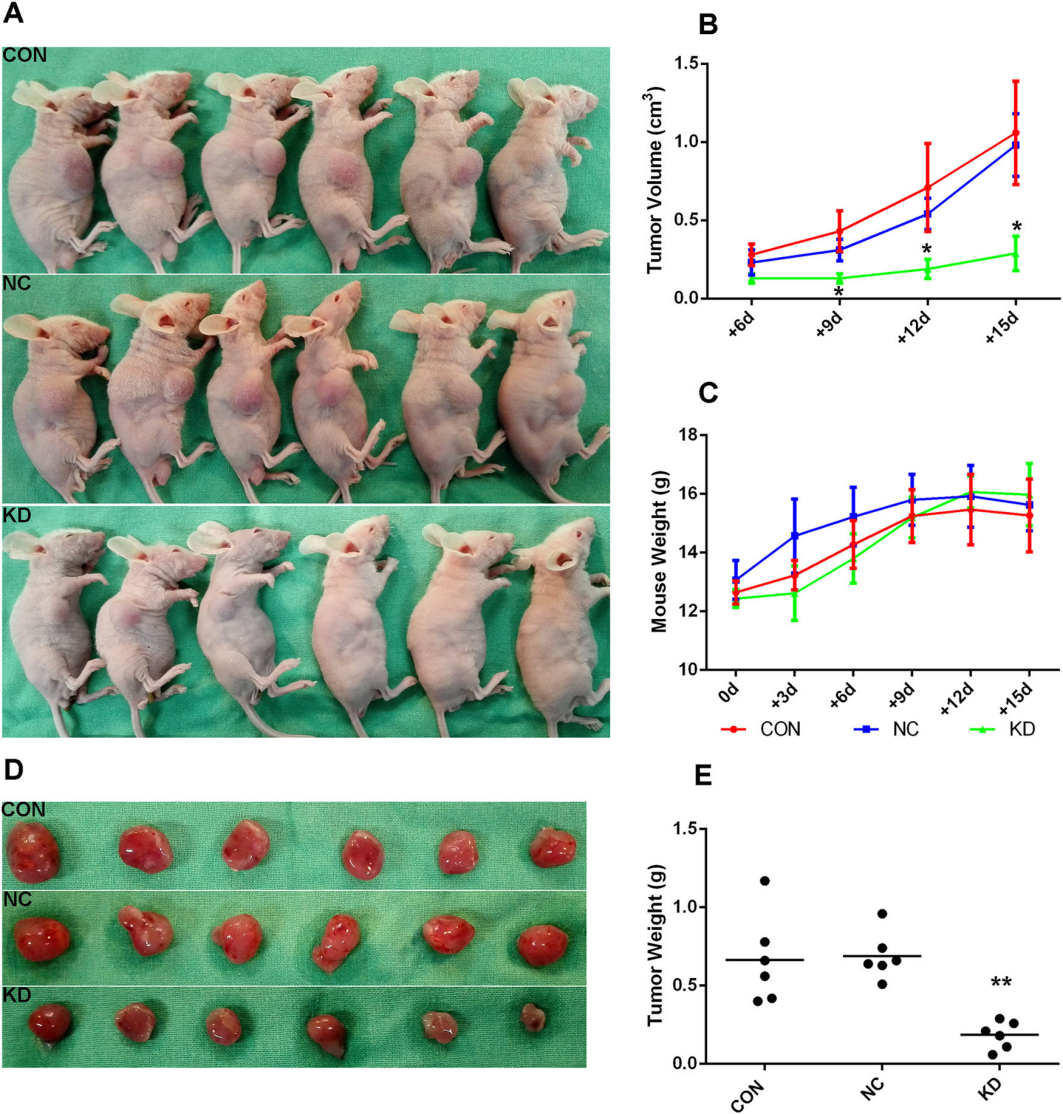
Silencing MTDH slowed down the growth of xenografts in vivo. Xenograft tumours on the nude mice (**a**), tumour volume in KD group was smaller (**b**), weight loss was observed in some mice starting 9 days after transplantation (**c**), tumours separated from the nude mice (**d**), tumour weight in KD group was smaller (**e**). **p < 0.01, *p < 0.05, compared with the CON group.

As mentioned above, silencing MTDH could inhibit the proliferation, induce the apoptosis and arrest the cell cycle of Jurkat cells in vitro. To further investigate the effect of silencing MTDH on the growth of Jurkat cells in vivo, the same number of Jurkat cells (1 × 10^7^ cells per mouse, including the CON, NC and KD groups) were transplanted into three groups of nude mice by subcutaneous injection to produce a leukaemia-bearing model. Appetite, behaviour and spirits were maintained in all mice, but weight loss was observed in some mice starting 9 days after transplantation (Fig. 6c). During the 15-day observation period, all mice formed tumours (Fig. 6a, d). However, the volume and weight of tumours in the KD group were significantly smaller than those in the CON group (*p* < 0.05), and no significant difference between the CON group and NC group was noted (*p* > 0.05) (Fig. 6b, d, e).

**Fig. 7:**
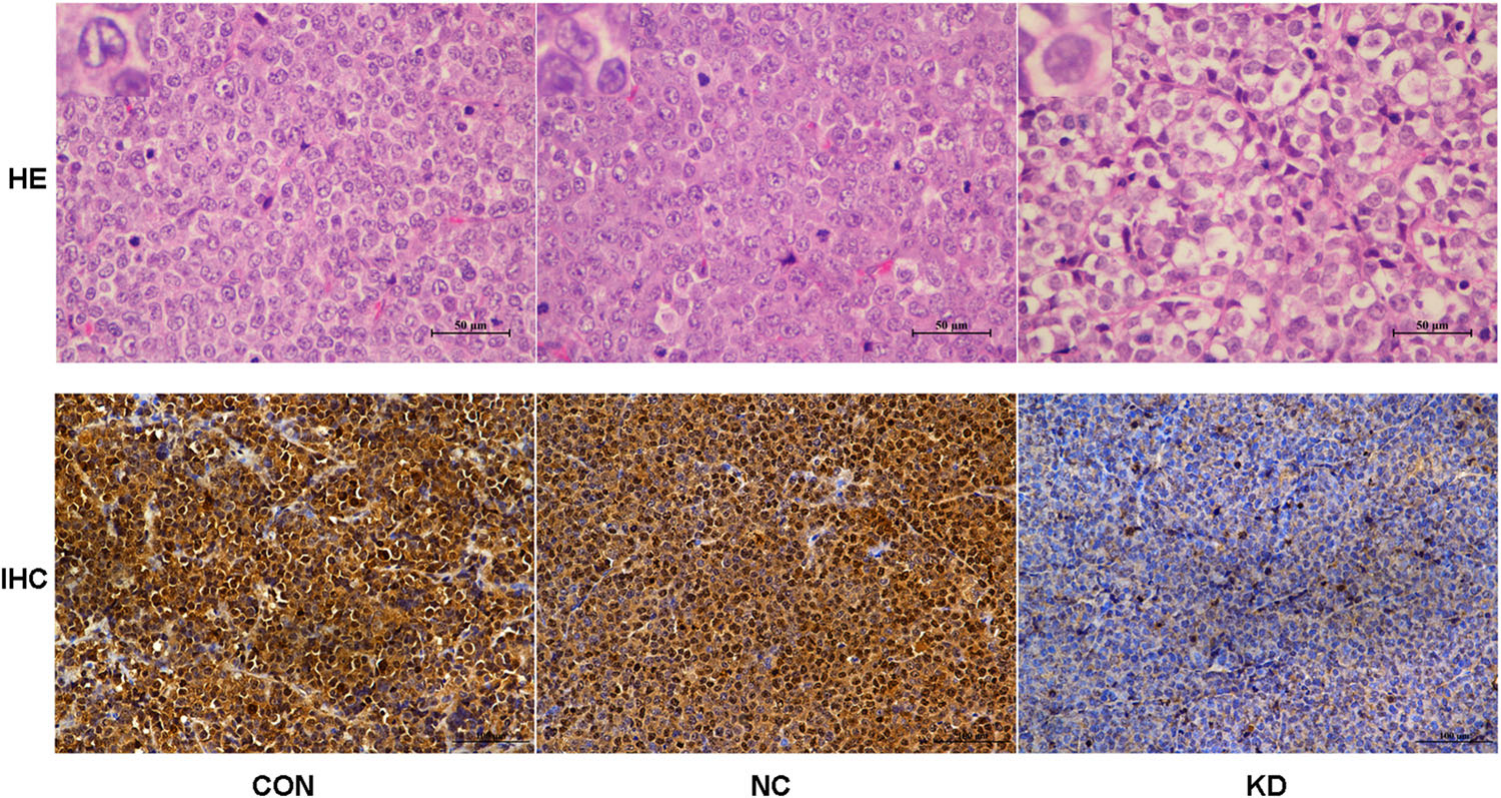
HE staining and IHC staining of Ki67 expression in the transplanted tumour tissues. HE staining (original magnification ×400) revealed that compared with the KD group, the CON group and NC group had more disordered cell arrangement and higher heterogeneity and cell division ability. IHC staining (original magnification ×200) showed that Ki67 expression was significantly reduced in the KD and NC groups compared with the CON group.

Fifteen days after transplantation, all of the nude mice were anaesthetized and sacrificed to dissect the xenograft tumours. The morphology and the expression of Ki67 in the transplanted tumour tissues were observed by microscopy after staining with HE and antibodies. Leukaemia cells in the CON and NC groups were disordered and showed more atypia and mitotic activity than those in the KD group. The expression of Ki67 in the CON and NC groups was significantly higher than that in the KD group (*p* < 0.01), while no difference was identified between the CON group and NC group (*p* > 0.05) (Fig. 7). These results were consistent with the in vitro data, suggesting that MTDH knockdown could slow the growth of Jurkat cells in vivo.

## Discussion

Previous research has shown that mPGES-1/PGE2 is highly expressed in a variety of malignant tumours, including acute leukaemia^5,20–22^, and is involved in regulating multiple signalling pathways and molecules^23^. However, few studies have reported the role of mPGES-1/PGE2 in T-ALL. In a previous study, we found for the first time that mPGES-1 and PGE2 are highly expressed in myeloid cell lines (K562, HL-60), a T-cell leukaemia cell line (Jurkat) and a B-cell leukaemia cell line (Raji) and that mPGES-1/PGE2 plays a partial role in regulating cell proliferation, apoptosis and cell cycle^19^. The current study is a deeper exploration of our previous study. In this study, we demonstrated that PGE2 could activate the cAMP-PKA pathway by binding to the EP3 receptor, subsequently inducing an augmentation of CREB expression and phosphorylation, and further increasing the expression of MTDH.

PGE2 has been proven to regulate downstream signalling pathways, such as cAMP-PKA, PI3K/AKT and MAPK/P38, by binding to EP receptors on the surface of the cell membrane^24^. The EP3 receptor is different from other EP receptors due to its different C terminus^25^. PGE2 binds to the G-protein-coupled receptors Gi, Gs, and G13 of EP3 and then activates or inhibits the cAMP/PKA-CREB pathway^26^. Kashiwagi et al.^27^ reported that downregulation of EP3 could promote prostate carcinogenesis by regulating AR expression. Other reports showed that EP3 was involved in the malignant phenotype of lung adenocarcinoma cells when stimulated by PGE2, and an EP3-specific inhibitor could block cell growth and Src activation^28^. These results indicate that the EP3 receptor may be an anticancer factor or an oncogenic factor, and its different roles in tumours may be related to the expression of different subtypes of the EP3 receptor. In the present study, we found that EP3 was a tumorigenic factor and exerted a crucial role in the regulation of mPGES-1/PGE2 in T-ALL progression. Inhibiting the EP3 receptor induced a reduction in the expression of cAMP, p-CREB, CREB and MTDH, which suggests that mPGES-1/PGE2 regulates the cAMP/PKA-CREB pathway at least partially through the EP3 receptor.

Published studies found that activation of the cAMP/PKA pathway increases the level of p-CREB, but not CREB, and the subsequent transcription of over 5000 downstream genes^29,30^. However, in our study, inhibiting the cAMP/PKA pathway reduced both CREB expression and phosphorylation. We speculate that the pathway may affect the expression of p-CREB by changing the level of CREB in Jurkat cells. The results are inconsistent with published studies. A recent study demonstrated that precise acupuncture at MTrPs combined with (or without) static stretching could inhibit the expression levels of CREB and p-CREB, while no further explanation was mentioned^31^. Whether there are other factors affecting the levels of CREB and p-CREB in Jurkat cells is unclear.

MTDH, also known as AEG1, is an oncogene that is considered to be an independent predictive factor for survival in various kinds of tumours. In some preclinical studies, MTDH-based DNA vaccines have been shown to induce the antitumour function of cytotoxic T lymphocytes and CD8+ T cells, and treatment with these vaccines inhibits tumour growth and metastasis in prostate cancer and enhances chemosensitivity to paclitaxel with minimal adverse effects^32,33^. In this study, MTDH was highly expressed in either T-ALL primary cells or Jurkat cells and expressed at low levels in HC-PBMCs. Silencing MTDH inhibited the proliferation and induced the apoptosis of T-ALL cells in vitro. Moreover, knockout of the MTDH gene significantly inhibited the growth of T-ALL xenograft tumours in nude mice. These results strongly suggest that MTDH is closely related to the development of T-ALL. Our results are in accordance with those of Yin et al.^34^ and Lee et al.^35^ in studies of other tumours.

However, the regulatory mechanism of MTDH is diverse. Studies have reported that several miRNAs, such as miR-98, miR-182-5p, miR-36645p and miR-384, could exert antitumour effects by targeting MTDH^36–39^. Sarkar et al.^40^ revealed that MTDH acted as a bridging factor by connecting NF-κB and CREB binding protein in malignant gliomas. In our study, inhibiting mPGES-1/PGE2/EP3 decreased the activity of the cAMP/PKA pathway and reduced the expression of p-CREB, CREB and MTDH, suggesting that mPGES-1/PGE2/EP3 may be located upstream of MTDH. Furthermore, the agonist of the cAMP-PKA pathway, forskolin, increased the expression of p-CREB, CREB and MTDH, while the inhibitor of the cAMP-PKA pathway, H89, had the opposite effect on the expression of p-CREB, CREB and MTDH, indicating that MTDH may be regulated by the cAMP-PKA pathway. By applying a dual-luciferase assay, we found that overexpression of CREB activated MTDH transcription.

MTDH exerts its function by promoting cancer progression, metastasis and chemoresistance by interacting with its downstream molecules and proteins, such as NF-kappaB, PLZF, Rrs1, beta-catenin and ubinuclein^40,41^. A clinical study revealed that MTDH was highly expressed in DLBCL tissues and may promote tumorigenesis via the Wnt/β-catenin pathway^42^. Another study found 104 (80.62%) T-cell-NHL tissues exhibited cytoplasmic MTDH immunostaining, and the mechanism may be associated with the promotion of survivin and Bcl-2/Bax protein expression and MMP-2/-9 activity^43^. However, there are limited reports on the role of MTDH in leukaemia. Only one team reported that MTDH is overexpressed in CLL and that this overexpression is closely related to patients’ clinical staging and is considered a promoter of the Wnt pathway^44,45^. In this study, we were not able to clarify the tumour-promoting mechanism of MTDH. The relationship between MTDH and treatment response and outcome of patients with T-ALL is still unknown. Further research is warranted.

**Fig. 8:**
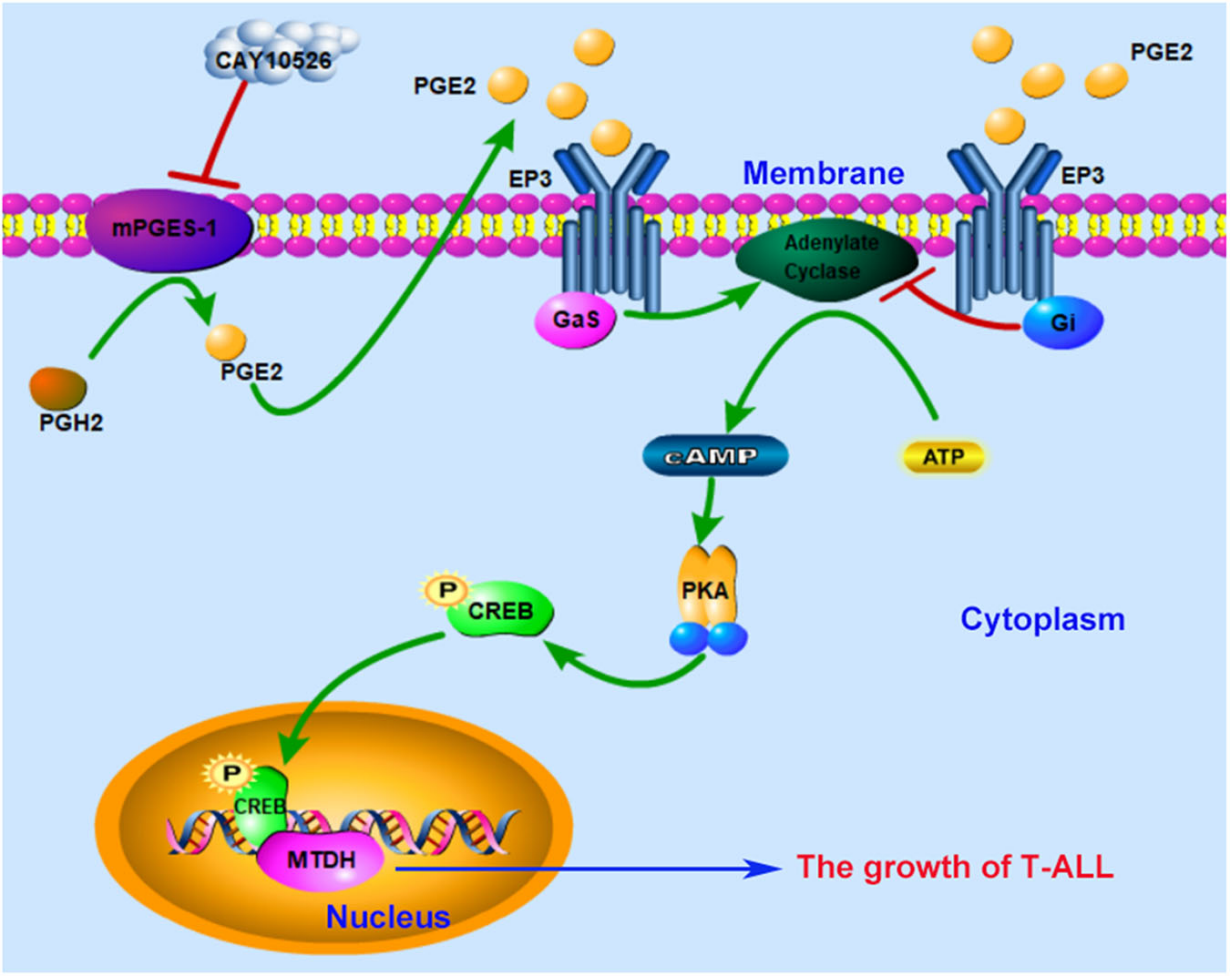
A model illustrated the possible mechanism by which mPGES-1/PGE2 regulated MTDH expression via the EP3/cAMP/PKA/CREB pathway in T-ALL.

In summary, we demonstrate for the first time that mPGES-1/PGE2 can activate the cAMP/PKA pathway, at least partially via the EP3 receptor, and regulate CREB expression and phosphorylation, subsequently increasing the expression of MTDH and improving the tumorigenesis and development of Jurkat cells in vitro and in vivo (Fig. 8). Our results suggest that targeting MTDH and its regulatory pathway could be a promising strategy for the treatment of T-ALL.
